# The Prognostic Long-Term Impact of Chronic Obstructive Pulmonary Disease and Postoperative Mucostasis in Patients with Curatively Resected Non-Small Cell Lung Cancer

**DOI:** 10.3390/cells12030480

**Published:** 2023-02-02

**Authors:** Joerg Lindenmann, Melanie Fediuk, Nicole Fink-Neuboeck, Iurii Mykoliuk, Elisabeth Taucher, Martin Pichler, Josef Smolle, Freyja Maria Smolle-Juettner

**Affiliations:** 1 Division of Thoracic Surgery and Hyperbaric Surgery, Department of Surgery, Medical University of Graz, 8036 Graz, Austria; 2 Division of Pulmonology, Department of Internal Medicine, Medical University of Graz, 8036 Graz, Austria; 3 Division of Oncology, Department of Internal Medicine, Medical University of Graz, 8036 Graz, Austria; 4 Department of Experimental Therapeutics, The UT MD Anderson Cancer Center, Houston, TX 77030, USA; 5 Institute of Medical Informatics, Statistics and Documentation, Medical University of Graz, 8036 Graz, Austria

**Keywords:** mucostasis, chronic obstructive pulmonary disease, non-small cell lung cancer, curative resection, prognosis, overall survival, tumor-free survival, cancer specific survival

## Abstract

Chronic obstructive pulmonary disease (COPD) serves as risk factor for the development of lung cancer and seems to have a prognostic impact after surgery for non-small cell lung cancer (NSCLC). The aim was to investigate the impact of COPD and postoperative mucostasis on the long-term survival after resected NSCLC. We retrospectively reviewed the data from 342 patients with curatively resected NSCLC. The prognostic long-term impact of COPD and postoperative mucostasis on overall survival (OS), recurrence free survival (RFS) and cancer specific survival (CSS) was calculated using univariable and multivariable Cox regression analyses. We found that 52.3% suffered from COPD and 25.4% had postoperative mucostasis. COPD was significantly more common among smokers (59.9%) compared with non-smokers (21.3%), (*p* < 0.001). There was a significant relationship between COPD and postoperative mucostasis (*p* = 0.006) and between smoking and mucostasis (*p* = 0.023). Patients with postoperative mucostasis had a significantly worse OS (*p* < 0.001), RFS (*p* = 0.009) and CSS (*p* = 0.008). The present analysis demonstrated that postoperative mucostasis, but not COPD, was associated with both worse short- and long-term outcomes for OS, RFS and CSS in curatively resected NSCLC.

## 1. Introduction

Although there have been evolving advances in the diagnosis and treatment of non-small cell lung cancer (NSCLC) in the past decades, the understanding of this multifactorial carcinogenesis is still under debate. However, lung cancer remains one of the leading causes of cancer deaths worldwide with an estimated 1.8 million deaths per year [1].

For this reason, accurate prognostication of long-term clinical outcome after curative surgery is of utmost importance for identifying patients at higher risk of recurrence and death [2]. Currently, the TNM staging system of the International Union Against cancer (UICC) has been widely used to predict prognosis according to the local, regional, and distant extent of the tumor. However, some authors highlight that the currently used TNM staging system might insufficiently capture patient prognosis. Importantly, it has been shown that recurrence and survival outcomes even differ within patients of the same tumor stage and after administration of the same treatment [3,4,5]. In this context, the established prognosticators after curative surgery for NSCLC are still under debate focusing on the development of new reliable prognostic parameters.

In the course of the ongoing search for appropriate prognostic parameters the impact of chronic inflammation on the development of lung cancer has been investigated. In this context, chronic obstructive pulmonary disease (COPD), listed by the World Health Organization (WHO) as the third leading cause of death worldwide, accounting for 3.23 million deaths in 2019 [6], has not only shown to serve as a major risk factor for the development of lung cancer regardless of smoking status [7] but, moreover, seems to have a prognostic impact on the survival of patients after curative resection.

It is well known that COPD involves chronic bronchitis, emphysema and small airway disease resulting in an irreversible destruction of lung parenchyma followed by impaired breathing. Long-term chronic inflammation is stimulated by the release of pro-inflammatory mediators by macrophages, and irritation of the airway epithelium causes mucus hypersecretion; furthermore, tissue remodeling of the small airways as well as hyperplasia of goblet cells and smooth muscle hypertrophy leads to decreased mucociliary clearance [8].

The severity of COPD has already been associated with the level of infiltration by inflammatory cells (e.g., macrophages, CD^4+^ and CD^8+^ T cells, neutrophils, dendritic cells and B cells) therefore suspecting a causal correlation between chronic inflammation and lung cancer. There is broad evidence that the higher the risk of carcinogenesis the longer the inflammation persists, even though the complex molecular interactions still remain uncertain [9,10].

The presence of COPD also seems to facilitate the development of lung cancer in never-smokers, especially in women, supporting the association between chronic inflammation, DNA damage, mutations and eventually cell growth, proliferation and carcinogenesis [7].

However, tobacco smoke is a main risk factor for the development of both COPD and lung cancer respectively. With its mixture of numerous toxic and carcinogenic compounds smoking does not only induces chronic inflammation but also affects the innate as well as the adaptive immune response in various pathways [11].

Although the correlation between COPD and lung cancer highly applies to smokers, with a 4.5-fold risk for lung cancer in long-term smokers [10], approximately one fourth to one third of COPD cases and about 15–25% of lung cancer cases concern never-smokers. Up to now lung cancer remains a thoracic malignancy caused by multiple factors whose genesis cannot be entirely explained by known risk factors, including tobacco smoking, environmental smoke, occupational exposure to carcinogens, radon gas, air pollution and genetic susceptibility, so far [7,11].

Apart from that, impaired mucociliary clearance has been identified as another possible risk factor in the development of both chronic bronchitis and bronchial carcinoma, respectively. Mucus hypersecretion, either through elevated mucin synthesis or mucus secretion, combined with possible innate errors of mucociliary clearance, results in mucostasis as well as mucus plugging the airways [12]. It has already shown that patients with lung cancer and smokers with chronic bronchitis have a significantly lower mucociliary clearance, with a more delayed clearing phase of the central bronchial tree rather than of the peripheral airways [12]. Interestingly, the mucociliary clearance rate seems to be even lower in patients with lung cancer and subsequent complications (e.g., infection, atelectasis and/or pleural effusion) compared to those cancer patients without complications [12].

To date there have been quite a few datasets investigating the association between COPD and survival in both smokers and never-smokers with NSCLC after curative resection [13,14]. To our best knowledge, the additional possible prognostic impact of postoperative mucostasis has not been addressed so far.

Therefore, the aim of this study was to investigate the impact of both COPD and postoperative mucostasis on the long-term survival in patients with curatively resected NSCLC.

## 2. Materials and Methods

We retrospectively analyzed 342 consecutive patients with NSCLC operated with curative intent between 01/2003 and 12/2007.

The inclusion criteria were male and female patients aged over 18 years with pathologically confirmed stage I-IIIB NSCLC after curative resection. Exclusion criteria were tumor stage greater than UICC stage IIIB and every type of sublobar resection. All patients had undergone preoperative spirometry and symptom limited cardio-pulmonary exercise test. COPD was defined as a preoperative ratio of FEV_1_ (forced expiratory pressure in 1 s) to FVC (forced vital capacity) of <70%, according to the recent GOLD guidelines (Global Initiative for Chronic Obstructive Lung Disease). Those postbronchodilator values were based on spirometry performed by qualified anesthesiologists following the criteria for the standardization of pulmonary function tests recommended by the American Thoracic Society/European Respiratory Society (ATS/ERS) Task Force as reported previously [9]. For the sake of completeness, it has to be mentioned that those patients suffering from COPD were all assigned to the bronchitis phenotype.

In the course of the pre-operative functional evaluation, the patient‘s smoking status was documented in the patient’s medical records. Passive or secondhand smoking was not assessed routinely and was therefore not available for further evaluation. During the retrospective data collection, documented active smokers and non-smokers were included in this clinical analysis. However, smoking was defined as the current and regular inhalation of the smoke of burning tobacco mainly encased in cigarettes.

Postoperative mucostasis was defined as the accumulation of mucus in the airways with subsequent mucoid obstruction of the bronchial opening of the lung segments, the lobes, the main bronchi or the trachea, respectively. In the postoperative period, the clinically suspected presence of mucostasis was confirmed endoscopically. In this regard, flexible fiber optic bronchoscopy was performed from time to time to remove viscous airway secretions with endoscopic application of bronchial toilette enabling both efficient and sustainable tracheo-bronchial clearance.

Basically, in severe cases of excessive mucostasis, supportive endoscopy may be required in the very early postoperative period (i.e., still in the operating room) in order to facilitate the tracheo-bronchial clearance. With flexible fiber optic bronchoscopy performed from time to time, selective removal of retained viscous airway secretions with application of bronchial toilette is done. In some cases, additional administration of continuous positive airway pressure (CPAP) at the intensive care or intermediate care unit may be required. While maintaining these supportive respiratory measures, continuous decrement of the postoperative mucostasis can be achieved.

Among the applied surgical procedures, patients had lobectomy, bi-lobectomy, bronchoplastic sleeve-resection or pneumonectomy with complete mediastinal lymph node dissection performed by board-certified thoracic surgeons. In those patients with early stage NSCLC without suspicion of mediastinal lymph node involvement, lobectomy was done using the minimal invasive approach known as thoracoscopic lobectomy or VATS lobectomy. In case of locally advanced tumor stage and/or neo-adjuvant chemotherapy requiring extended and/or bronchoplastic resection, conventional open surgical resection by antero-lateral or postero-lateral thoracotomy was performed.

Sublobar resections (wedge resections and segmental resections) have been shown to be connected with higher rates of postoperative tumor recurrence and decreased survival compared with standard resection procedures and were therefore excluded from this retrospective evaluation [15,16,17].

Tumor staging was done according to the current tumor-node-metastasis (TNM) classification defined by the Union for International Cancer Control (UICC International Union Against Cancer, TNM Classification of Malignant Tumors, 8th edition) [18].

Overall survival (OS) was defined as the time from the date of surgery to the date of death-from-any-cause. Recurrence free survival (RFS) was calculated from the date of surgery to the date of diagnosis of tumor recurrence. Cancer specific survival (CSS) was determined from the date of surgery to the date of death after tumor recurrence.

### 2.1. Postoperative Follow-Up Schedule

Regardless of adjuvant therapy, all patients were followed up according to a regular schedule with visits every 3 months during the first two years, every 6 months until the 5th year, and every 9 to 12 months until the 10th year, with yearly follow-up thereafter.

The investigations comprised chest roentgenograms and clinical investigations at every visit. With resected NSCLC stage I and II, thoracic CT scans were done every 6 months during the first three years, followed by further scans every 12 months. In patients with operated NSCLC stage III, thoracic CT scans were scheduled every 3 months during the first two years, and every 6 months during the third year after surgery, followed by scans at annual intervals starting with the 4th postoperative year. Those patients with resection of tumors affecting the central airways and subsequent bronchoplastic reconstruction underwent additional surveillance bronchoscopy every 6 months during the first two years and every 12 months beyond that. In this subgroup of patients CT scans were scheduled every 3 months during the first two years, and every 6 months during the third year after surgery, followed by scans at annual intervals starting with the 4th postoperative year as previously reported [19].

### 2.2. Data Management

The patient specific data were collected prospectively in the database of our hospital and retrospectively extracted for statistical evaluation as previously mentioned [19]. The required follow-up data were retrieved from the Regional Health Care System database (openMEDOCS; developed by the Steiermärkische Krankenanstaltengesellschaft, Graz, Austria). If a patient had not presented for follow-up, the respective primary health care provider was contacted for further information. If the patient was suspected to have died, we queried the Civil Registry. The causes of death were recorded. No patient was lost to follow-up. All cases were followed up through January 2022 or until death.

### 2.3. Statistical Analysis

Statistical analysis was performed using STATA version 17 (Stata Corp, College Station, TX, USA). Besides descriptive statistics (absolute and relative frequency, mean, standard deviation), a chi^2^ test and *t*-test were applied whre appropriate. Survival rates were calculated by life table analysis, and statistical comparisons of survival functions were assessed by Cox proportional hazard model in an univariable and a multivariable approach. Univariable survival analysis was performed with Kaplan–Maier survival curves and Mantel–Haenszel log-rank test. Furthermore, propensity score analysis (inverse probability of treatment weights) were applied. *p* < 0.05 was considered to indicate statistical significance.

## 3. Results

The collective consisted of 225 males (65.8%) and 117 females (34.2%). The vast majority of those patients were smokers (274; 81.8%), 181 patients (52.9%) suffered from COPD and 87 patients (25.4%) developed postoperative mucostasis. Within this mucostasis group, postoperative flexible fiber optic bronchoscopy was done in every patient. A total number of 250 bronchoscopies were therefore performed (range 1–14; median 2.0 per patient). 121 toilets of the bronchial tree were done (range 1–12; median 1.0 per patient).

Among this mucostasis group, only seven patients were connected with pulmonary infection corresponding to pneumonia. Altogether, 13 patients developed postoperative pneumonia whereas in the remaining six cases preceding mucostasis could not be detected (*p* = 0.016). Table 1 shows the comparison of these demographic clinical parameters mentioned above between the non-COPD and COPD groups, the non-mucostasis and mucostasis groups and the non-smoker and smoker groups, respectively.

Among the surgical procedures, there were 315 (92.1%) lobectomies or bi-lobectomies (thereof 26 [7.6%] using sleeve-resection technique) and 27 (7.9%) pneumonectomies. Regarding the postoperative histological evaluation, adenocarcinoma was the dominating subtype in 140 patients (40.9%), followed by squamous cell carcinoma in 112 patients (32.7%), whereas 165 patients (48.2) had G3, 135 (39.5%) G2 and only 42 (12.3%) G1. The pathological staging yielded T1 in 186 (54.4%), T2 in 128 (37.4%), T3 in 18 (5.8%) and T4 in 7 (2%) cases; N0 was found in 191 (55.8%), N1 in 93 (27.2%) and N2 in 58 (17%) of patients. Considering these two parameters, UICC staging revealed stage I in 182 patients (53.7 %), stage II in 85 patients (25.1 %) and stage III in 72 patients (21.2 %). A statistically significant relationship could be observed for COPD (*p* = 0.040), but not for mucostasis (*p* = 0.682) or smoking (*p* = 0.586). There were 44 patients who had preoperative induction chemotherapy, resulting in complete pathological response in three of them. Adjuvant chemotherapy was scheduled in 93 cases, adjuvant chemo-radiotherapy in 11 and adjuvant radiotherapy in 34. Table 2 shows the comparison of these tumor-related clinical parameters mentioned above between the non-COPD and COPD groups, the non-mucostasis and mucostasis groups and the non-smoker and smoker groups, respectively.

In this context, COPD was significantly more common in the group of smokers (163/272; 59.9%) compared with the non-smokers (13/48; 21.3%), (*p* < 0.001). There was a significant relationship between COPD and postoperative mucostasis (*p* = 0.006) and between smoking and mucostasis (*p* = 0.023). In addition, there was a statistically significant relationship between the male sex and COPD (*p* < 0.001), mucostasis (*p* = 0.042) and smoking (*p* < 0.001), respectively. There was a statistically significant relationship between histology and COPD (*p* = 0.015) as well as smoking (*p* < 0.001).

The median follow-up time for the OS collective was 149 months (range: 125–184 months) and for the RFS collective 151 months, respectively. Postoperative tumor recurrence could be observed in 175 patients (51.2%). The vast majority (41.2%) developed distant recurrence whereas only 34 patients (9.9%) had loco-regional recurrence. However, 25 patients (7.3%) had a second primary lung cancer (median interval after resection of the primary NSCLC, 83 months; ranged 15–155 months). The overall mortality rate for the entire follow-up period was 71.6% (245/342). 157 patients (45.9%) died of lung cancer, 22 died of tumors other than bronchial carcinoma and 66 patients died of causes other than neoplasia, thereof six in the perioperative course (1.75%). However, only three patients (0.9%) died of severe COPD whereas nine patients (2.6%) died from postoperative pneumonia. Among this second subgroup seven patients had preceding mucostasis. The details are given in Table 3. There was a statistically significant relationship between mucostasis and survival in both univariable and multivariable analysis (Table 4).

**Figure 1:**
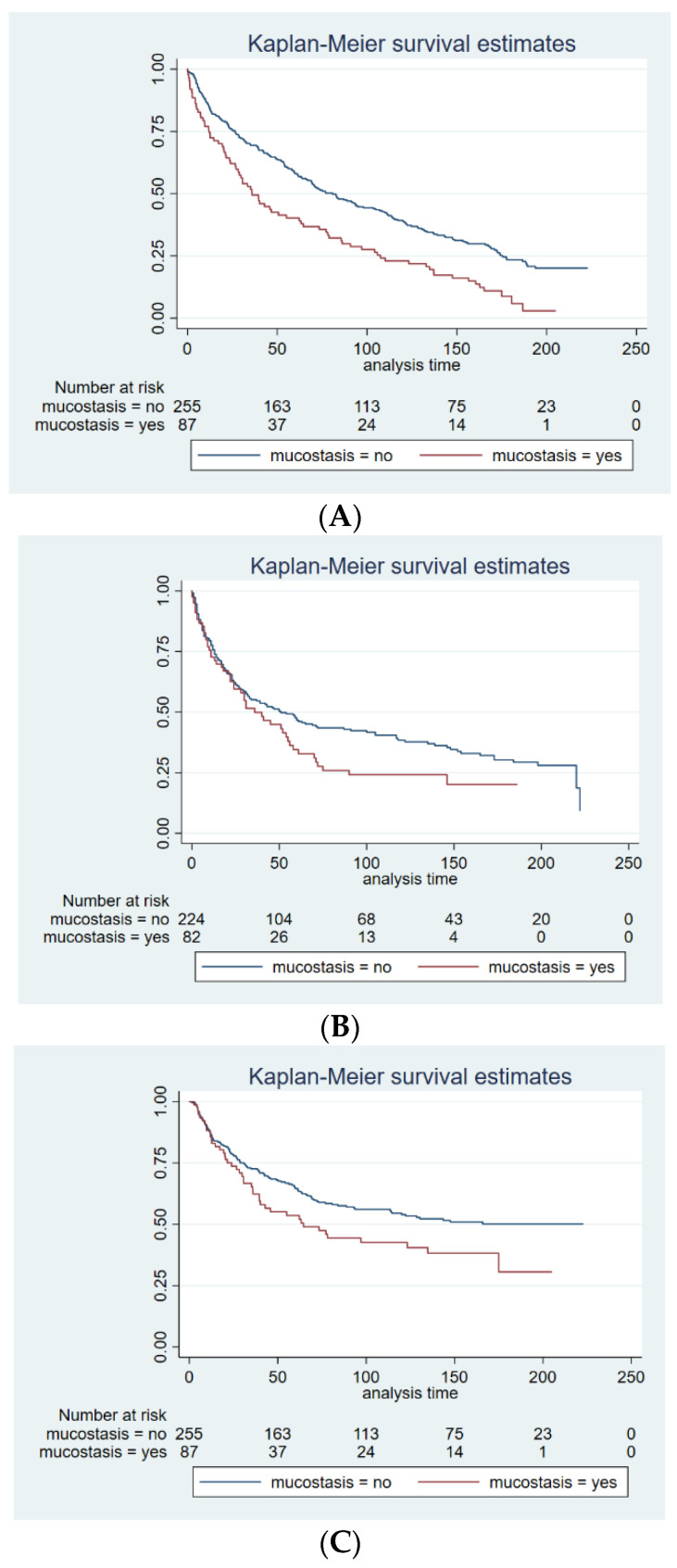
(**A**) Kaplan–Meier curves comparing the 10-year survival rates between those patients with postoperative mucostasis and those without among the collective of 342 patients undergoing curative resection for NSCLC showing OS (*p* < 0.001). Abbreviations: OS: overall survival, NSCLC: non-small cell lung cancer. (**B**). Kaplan–Meier curves comparing the 10-year survival rates between those patients with postoperative mucostasis and those without among the collective of 342 patients undergoing curative resection for NSCLC showing RFS (*p* = 0.009). Abbreviations: RFS: recurrence free survival, NSCLC: non-small cell lung cancer. (**C**) Kaplan–Meier curves comparing the 10-year survival rates between those patients with postoperative mucostasis and those without among the collective of 342 patients undergoing curative resection for NSCLC showing CSS (*p* = 0.008). Abbreviations: CSS: cancer specific survival, NSCLC: non-small cell lung cancer.

Patients with postoperative mucostasis had a significantly worse OS (*p* < 0.001), RFS (*p* = 0.009) and CSS (*p* = 0.008) as shown in Figure 1A–C. The 1-, 3-, 5- and 10-year-OS was 62%, 43%, 37% and 12% for patients with mucostasis compared to 76%, 65%, 53%, 27% for non-mucostasis patients. For the same subgroup the CSS was 74%, 55%, 48% and 27% compared to 78%, 68%, 57% and 41%. For the RFS 66%, 48%, 36%, 29% compared to 67%, 56%, 49%, 36% were detected.

There was no statistically significant relationship between COPD and survival and smoking and survival, neither in univariable nor in multivariable analysis (Table 4). When the potential relationship of COPD, mucostasis, and smoking on OS, RFS and CSS was additionally evaluated using propensity score analysis (inverse probability of treatment weights), the results were only marginally different from those obtained by conventional analysis. As shown in Table 4, age (*p* < 0.001), BMI (body mass index) (*p* = 0.005), tumor stage (*p* < 0.001) and pre-operative treatment (*p* = 0.002) were additional independent predictive factors for OS. For CSS age (*p* = 0.023), BMI (*p* = 0.021), histological subtype (*p* = 0.013), tumor stage (*p* < 0.001) and pre-operative treatment (*p* = 0.006) served as additional independent prognostic parameters. For RFS BMI (*p* = 0.018), histological subtype (*p* = 0.005), tumor stage (*p* < 0.001) and pre-operative treatment (*p* = 0.017) were additional independent parameters (Table 4).

## 4. Discussion

This retrospective clinical study demonstrates that postoperative mucostasis has been associated with both worse short- and long-term outcomes for OS, RFS and CSS in patients undergoing curative resection for NSCLC during 10-year follow-up whereas preoperative COPD was not significantly associated with postoperative mortality and prognosis, respectively. To our best knowledge the current study is the first clinical study which has investigated the impact of postoperative tracheo-bronchial mucostasis on the long-term survival of patients with curatively resected NSCLC.

Basically, airway mucus is an aqueous solution consisting of lipids, proteins and mucins. These mucins are produced by the epithelial goblet cells and submucosal glands of the tracheo-bronchial airways.

Airway mucus, which consists of two layers, has an important cleaning and transport function within the tracheo-bronchial system. The upper gel layer encases inhaled airborne particles and pathogens which are moved centrally by the epthelial cilia. Beneath, there is a waterier layer that lubricates cilia and ensures mucus spread all over the airway epithelium [8,20]. Dysfunction of the mucis synthesis or regulation as well as the function of the cilia result in impairment of the mucociliary clearance with subsequent breathing disability.

However, postoperative mucostasis remains a crucial condition in patients after thoracic surgery, in particular after anatomical lung resection. Among the collective of smokers suffering from COPD, postoperative mucostasis may increase considerably which may represent an additional therapeutic challenge for the confronted clinician. In order to overcome this condition, muco-active therapies including expectorants, inhalations, mucolytics and respiratory physiotherapy have to be initiated.

Already in the preoperative setting we start these supplementary therapeutic interventions to prevent postoperative mucostasis where possible. In this context, every patient who has been admitted to our division for lung surgery will be visited daily by our physiotherapist performing pre-operative breathing exercises for effective coughing. Especially in those patients who are prone to mucostasis due to COPD and smoking their bronchodilator medication is optimized and the breathing exercises are intensified tailored to their specific needs.

In the postoperative setting these therapeutic interventions as mentioned above are initiated as soon as possible in particular in this very subset of patients. Already several hours after surgery these patients start inhalation therapy with a bronchodilator (i.e., ipratropium bromide and fenoterol hydrobromide) combined with nebulized secretolytics (i.e., tyloxapol) three times a day. In order to relieve independent expectoration adjunctive mucolytic agents (i.e., N-acetylcysteine) are administered once a day. Intensified respiratory physiotherapy with breathing exercises and patient mobilization are performed additionally.

In this context, mucus hypersecretion is caused by progressive long-term inflammation of the lower airways in the course of chronic bronchitis. Both hypertrophy of the submucosal glands and hyperplasia of the goblet cells in the bronchial epithelium are leading to an excessive mucus production resulting in impaired mucociliary clearance [8]. Furthermore, due to consistent release of various inflammatory mediators by macrophages in response to cigarette smoke and other noxious agents [11], chronic irritation of the bronchial epithelium is induced resulting in tissue remodeling. In this context, airway fibrosis and smooth muscle hypertrophy develop which promote the continuous decrease of the mucociliary clerance rate [8]. Besides that, replacement of the columnar epithelium by squamous epithelium has been described. This so called squamous cell metaplasia contributes to the decreased mucociliary clearance and represents a considerable risk factor for the development of lung cancer, in particular squamous cell carcinoma, in those patients suffering from COPD [10,21].

Matthys et al. confirmed these findings by measurement of the mucociliary clearance, showing that the mucociliary clearance rate of patients with bronchial carcinoma is significantly lower compared to normal patients and compared to patients suffering from only chronic bronchitis [12].

It has been demonstrated that tobacco smoking induces neutrophils and activated macrophages to release various cytokines, inflammatory mediators and growth factors including TNF-α, TGF-β, EGFR, G-CSF, IL-1, IL-8 as well as oxygen radicals. As mentioned above, mucus secretion with subsequent mucostasis is considerably increased by this ongoing inflammatory condition contributing to the development of lung cancer [7,22,23]. This inflammatory mucus-related mechanism might be a possible explanation why postoperative mucostasis is associated with a higher risk for tumor recurrence as proven in the current study.

By the release of these oxygen radicals, chronic inflammation is induced, which may persist for decades even after smoking cessation [8,10]. This long time interval may be an explanation for the immanent risk of tumor recurrence within the lung itself, even after curative resection. Recent data which has been published by our group confirm this assumption. In this context, the development of a second primary lung cancer could be confirmed in 7.3% of all cases at a median time interval of 83 months after resection of the primary NSCLC.

In addition, we could demonstrate that postoperative tumor recurrence, which could be confirmed in 51.2% of all cases, largely determined the postoperative prognosis, also in patients with other accompanying tumors [19]. Whereas 45.9% of our patients died from the primary lung cancer, only 6.4% died from another malignancy and 19.3% died from other causes than malignancy as shown in Table 2. In this context, survival was significantly shorter in patients who died from primary lung cancer compared with patients who died of other tumors or none-tumor-related causes [19].

Moreover, the present data could show that the patient´s age serves as an independent prognostic parameter for the CSS. In this regard, we could observe an interesting aspect of the current study. The included patients were considerably younger showing up with a mean age of 63.6 years compared with recent cohorts [24].

According to the findings of the present clinical study BMI, tumor stage and neo-adjuvant chemotherapy have shown to serve as additional independent prognostic parameters (Table 4) which are favorably in accordance with corresponding data from the literature [25,26,27,28]. The present data attest that reduced BMI has a negative impact on postoperative OS, CSS and RFS, respectively. This is in line with data of a systematic review published by Wang et al. in 2018. They could demonstrate that lung cancer patients with a higher BMI have a longer survival than those with a lower BMI [25]. In contrast, recent data have shown that there might be no unanimous consensus on better survival for curatively resected lung cancer patients with increased BMI. Among 5088 patients undergoing minimally invasive surgery no statistically significant difference for RFS and OS could be found [24].

However, lung cancer patients with reduced BMI may show characteristic properties: they exhibit pre-existing co-morbidity including extensive smoking history, they present in a poor functional performance, they complain about continuous weight loss and they may suffer from catabolic status, respectively.

In this regard, inflammation-related mucus hypersecretion leads to impaired mucociliary clearance, mucostasis and development of viscous mucus clogging the airways. Along with the decreasecd BMI and the associated reduced muscle capacity for forceful and productive expectoration, the patient´s ability for breathing is depleted considerably.

As a consequence of this, the impaired mucociliary transport leads to longer residence time of inhaled and deposited agents. By the additional release of oxygen radicals, the perpetuation of this chronic inflammatory condition is initiated which may persist for many years even after smoking cessation [8,10] contributing considerably to the development of lung cancer [7,22,23], as mentioned above. Besides the reduced BMI, mainly the postoperative mucostasis represents an aggravating prognostic parameter contributing to reduced postoperative long-term survival which could be confirmed by the present data.

Interestingly, the present data shows that contrary to our assumption, pre-operative COPD does not serve as independent prognostic parameter for postoperative survival. Although COPD and lung cancer have shown to coexist in the majority of the cases [29], our data confirmed that there was no statistically significant impact of COPD on the RFS and CSS, respectively. Furthermore, COPD was not associated with higher mortality in either uni- nor multi-variate analysis. These findings are in line with the results obtained from other authors who could corroborate that COPD had no impact on the mortality of patients with NSCLC [29,30,31].

The reasons therefor may be speculative. Ytterstad et al. assumed that the majority of their NSCLC patients had moderate COPD and due to the short survival time, in case of advanced tumor stage, COPD might have no significant impact on survival [30]. Other authors supposed that in particular in patients with operated early stage NSCLC COPD tends to rise the mortality rate. In contrast, after inclusion of patients with all tumor stages, COPD might not affect the postoperative survival. This could explain this divergent finding together with a relatively small study population [29,31]. In this context, all authors endorsed that the retrospective study design using clinical records as data resource represents a potential limitation [29,30,31].

However, the data of the present study are in contradiction with similar clinical studies which could impressively confirm COPD as an independent prognosticator in patients with lung cancer after curative surgery [7,13,14,32,33,34,35,36,37]. The closed relationship between lung cancer and COPD might be the most important reason because both diseases share the same common risk factors [32,33]. The detailed mechanism through which COPD may influence the prognosis of lung cancer still remains ambiguous and needs to be clarified [32,36]. As mentioned above, COPD is accompanied by both local pulmonary and systemic chronic inflammation, which is responsible for increased DNA damage with subsequent molecular injury, repair and genetic errors. In this context, abnormal apoptosis and cell cycle regeneration as well as aberrant DNA methylation are described. As a consequence, this COPD-related inflammatory microenvironment produces oxidative stress which damages DNA resulting in tumor growth. These mechanisms considerably reduce treatment effects in lung cancer resulting in decreased survival and increased mortality, respectively [13,36]. Zhai et al. could validate this hypothesis by their clinical findings. Among 902 patients with operated early stage NSCLC this subgroup with co-existent COPD had both significantly worse OS and reduced RFS respectively [36]. Similar results could be obtained by Akamine et al. who could show among 548 operated NSCLC patients that those patients with COPD had a significantly worse RFS and CSS, respectively [14]. Regarding these inflammation-associated mechanisms mentioned above, it is obvious that persisting inflammation which is often seen in the vast majority of COPD may serve as a considerable stimulus for carcinogenesis resulting in decreased RFS and CSS and therefore increased mortality.

Beside the cancer-related increase of mortality in patients with COPD, we are able to identify a second reason for increased mortality in this subset of patients. In this context COPD-related pulmonary insufficiency has shown to serve as the main cause of non cancer-related deaths as previously reported [33].

Basically, patients with COPD are more prone to develop pneumothorax, pneumonia and chronic hypoxemia because of impaired distance of alveolar surface to the capillary endothelium and reduced area of gas exchange. Moreover, pulmonary atelectasis due to increased sputum and altered mucociliary clearance occurs more frequently in this subgroup of patients. These conditions have a strong impact on deterioration of patient´s pulmonary condition after surgery and in the further course considerable decline of pulmonary function is seen. Once lung function is compromised postoperatively, inflammatory changes accelerate the destruction of viable lung tissue. These alterations may be precedents to formation of pneumonia and exacerbation of COPD itself therefore causing higher morbidity and mortality. This is why lung cancer patients with COPD have a higher mortality rate due to postoperative respiratory failure and right heart failure, respectively [33]. In this context Lopez-Encuentra et al. investigated the effect of COPD in 2994 patients with operated lung cancer. They could show that COPD serves as prognostic factor and that there is a clear relationship between the severity of COPD and postoperative survival [37]. These data were supported by a meta-analysis recently published by Wu et al. who confirmed that NSCLC patients with co-existing COPD were associated with reduced survival rate and increased mortality rate [32].

Relating to the findings of the present study, we could corroborate the increased percentage of patients with co-existent COPD (54.3%) within the mortality group, which could be continued in the split subgroups. In the subset of tumor-related death (N = 179) 50.8% had COPD whereas in the group of non-tumor-related death (N = 66) 63.6% suffered from COPD. Due to the fact that disease-related mucostasis, which serves as a functional consequence of smoking and COPD, has an independent prognostic impact on survival in the current study, we might assume that both COPD and smoking would have an indirect influence on survival but however, without statistical significance.

Considering recent data in literature, squamous cell carcinoma seems to represent the most common histological subtype of lung cancer among European men with COPD [21,36] We were able to confirm this assertion in the present data. In the COPD-subgroup, 36.5% had squamous cell carcinoma whereas 33.7% had adenocarcinoma, although among the whole collective adenocarcinoma was the dominating histological subtype (40.9%). COPD-related diseases like chronic bronchitis and lung emphysema have shown to be linked with a 33% to 50% higher risk for NSCLC in male patients [7,38,39].

Our present data corroborates this statement showing that male sex was dominating in both the whole collective (65.8%) and in the COPD-subgroup (76.2%), respectively. Moreover, there was a statistically significant relationship between tumor histology and COPD as well as smoking.

The outstanding strength of the present clinical study represents the long lasting and close meshed follow-up schedule which has been applied in every single patient corresponding to the underlying postoperative tumor stage. This meticulous procedure which has been embedded in the interdisciplinary tumor board ensured a consistent follow-up until which allowed for an at least 10 years observation period for the collective as previously reported [19]. With respect to the present data it would be beneficial to take the postoperative mucostasis into consideration when the further onco-surgical follow-up has to be scheduled. In particular this subset of patients with COPD and smoking- related mucostasis seem to have a worse prognosis and are therefore at higher risk for tumor recurrence. They might benefit from an intensified follow-up treatment consisting of periodical CT scans and bronchoscopies which should be tailored to their individual needs.

However, there are some limitations in the present study which have to be addressed. First, the study was retrospective, observational and conducted at a single institution. Secondly, due to the heterogeneous collective of this study, we cannot rule out the presence of some residual confounding by factors that were not included in the analysis due to not being collected during data ascertainment. Further, this relatively large cohort of 342 patients involved all current potentially resectable tumor stages with the corresponding different kind of resections.

Due to these facts the heterogeneity of the collective was increased considerably. Thirdly, neither secondhand smoke exposure in non-smokers nor occupational history or air pollutant exposure history was available for data analysis, making it difficult to speculate how these missing variables may would have influenced the results of the present study.

Finally, the absence of molecular diagnosis for lung cancer has to be addressed. The determination of the current genetic markers and biomarkers has not been done in this clinical study. The epidermal growth factor receptor (EGFR) status and other current genetic markers have not been evaluated pre-operatively among this collective because with the beginning of the 21st century the EGFR mutation status and other current biomarkers were not determined routinely due to their novelty.

## 5. Conclusions

Regarding the findings of the present clinical study we are able to conclude that postoperative mucostasis remains a not trivial and beyond that independent prognostic parameter in patients with COPD and smoking history who had undergone curative resection for NSCLC. It might be a meaningful approach to incorporate this clinical parameter into the postoperative follow-up in particular in this subset of patients who seem to be at higher risk for tumor recurrence. Nevertheless, it is of importance to prevent the development of postoperative mucostasis at the earliest possible moment by initiating these prophylactic and mucolytic measures mentioned above. However, further prospective studies of larger populations may be needed to confirm our recent findings and to reveal the detailed mechanisms between the risk factors COPD, smoking, chronic inflammation and subsequent carcinogenesis.

## Figures and Tables

**Table 1:** Clinico-pathological characteristics of 342 patients undergoing curative resection for NSCLC focusing on demographic data. *p* values refer to *t*-test for continuous variables and to chi^2^ for categorical variables. Abbreviations: COPD: Chronic obstructive pulmonary disease, BMI: Body Mass Index, kg: kilogram.

	All Cases *n* = 342	Non-COPD *n* = 161 (47.1%)	COPD *n* = 181 (52.9%)	Significance	Non-Mucostasis *n* = 255 (74.6%)	Mucostasis *n* = 87 (25.4%)	Significance	Non-Smoker *n* = 68 (19.9%)	Smoker *n* = 274 (80.1%)	Significance
Age (years; mean)	63.6 ± 9.5	63.1 ± 9.7	64.1 ± 9.3	*t* = −0.98, *p* = 0.328	63.1 ± 9.7	65.2 ± 87.8	*t* = −1.85, *p* = 0.0651	67.8 ± 9.9	62.6 ± 9.1	*t* = 4.12, *p* = 0.000
Weight (kg; mean)	75.5 ± 14.3	74.4 ± 14.4	67.5 ± 14.1	*t* = −35, *p* = 1.0.178	75.1 ± 14.3	76.6 ± 14.4	*t* = −0.85, *p* = 0.3976	71.5 ± 11.4	76.4 ± 14.8	*t* = −2.56, *p* = 0.011
BMI (kg/m^2^; mean)	25.9 ± 4.0	25.9 ± 4.0	26.0 ± 4.0	*t* = −0.26, *p* = 0.795	25.8 ± 3.8	26.2 ± 4.5	*t* = −0.89, *p* = 0.3693	25.8 ± 3.5	25.9 ± 4.1	*t* = −0.19, *p* = 0.849
Gender										
Male	225 (65.8%)	87 (45.0%)	138 (76.2%)	chi^2^ = 18.67, *p* = 0.000	160 (62.8%)	65 (74.7%)	chi^2^ = 4.13, *p* = 0.042	27 (39.7%)	198 (72.3%)	chi^2^ = 25.66, *p* = 0.000
Female	117 (34.2%)	74 (46.0%	43 (23.8%)		95 (37.3%)	22 (25.3%)		41 (60.3%)	76 (27.7%)	
Smoking Status										
No	68 (19.9%)	50 (31.1%)	18 (9.9%)	chi^2^ = 23.83, *p* = 0.000	58 (22.8%)	10 (11.5%)	chi^2^ = 5.15, *p* = 0.023			
Yes	274 (80.1%)	111 (68.9%)	163 (90.1%)		197 (77.3%)	77 (88.5%)				
COPD										
No	161 (47.1%)				131 (51.4%)	30 (34.5%)	chi^2^ = 7.43, *p* = 0.006	50 (73.5%)	111 (40.5%)	chi^2^ = 23.83, *p* = 0.000
Yes	181 (52.9%)				124 (48.6%)	57 (65.5%)		18 (26.5%)	163 (59.5%)	
Mucostasis										
No	255 (74.6%)	131 (81.4%)	124 (68.5%)	chi^2^ = 7.42, *p* = 0.006				58 (85.3%)	197 (71.9%)	chi^2^ = 5.15, *p* = 0.023
Yes	87 (25.4%)	30 (18.6%)	57 (31.5%)					10 (14.7%)	77 (28.1%)	

**Table 2:** Clinico-pathological characteristics of 342 patients undergoing curative resection for NSCLC focusing on tumor-related data. *p* values refer to chi^2^ for categorical variables. Abbreviations: COPD: Chronic obstructive pulmonary disease, G: histological grading, T: T stage (tumor invasion), UICC: International Union against Cancer.

	All Cases *n* = 342	Non-COPD *n* = 161 (47.1%)	COPD *n* = 181 (52.9%)	Significance	Non-Mucostasis *n* = 255 (74.6%)	Mucostasis *n* = 87 (25.4%)	Significance	Non-Smoker *n* = 68 (19.9%)	Smoker *n* = 274 (80.1%)	Significance
Histology										
Adenocarcinoma	140 (40.9%)	79 (49.1%)	61 (33.7%)	chi^2^ = 8.34, *p* = 0.015	110 (43.1%)	30 (34.5%)	chi^2^ = 3.228, *p* = 0.199	43 (63.2%)	97 (35.4%)	chi^2^ = 17.55, *p* = 0.000
Squamous cell carcinoma	112 (32.8%)	46 (28.6%)	66 (36.5%)		77 (30.2%)	35 (40.2%)		13 (19.1%)	99 (36.1%)	
Other	90 (26.3%)	36 (22.4%)	54 (29.8%)		68 (26.7%)	22 (25.3%)		12 (17.7%)	78 (28.5%)	
Grading										
G1	42 (12.3%)	26 (16.2%)	16 (8.8%)	chi^2^ = 5.20, *p* = 0.074	33 (12.9%)	9 (10.3%)	chi^2^ = 0.49, *p* = 0.782	12 (17.7%)	30 (11.0%)	chi^2^ = 3.49, *p* = 0.175
G2	135 (39.4%)	65 (40.4%)	70 (38.7%)		101 (39.6%)	34 (39.1%)		29 (42.7%)	106 (38.7%)	
G3	165 (48.3%)	70 (43.5%)	95 (52.5%)		121 (47.5%)	44 (50.6%)		27 (39.7%)	138 (50.4%)	
Tumor size										
T0	0 (0.9%)	0 (0.0%)	3 (1.7%)	chi^2^ = 7.89, *p* = 0.096	2 (0.8%)	1 (1.2%)	chi^2^ = 3.37, *p* = 0.497	0 (0.0%)	3 (1.1%)	chi^2^ = 5.36, *p* = 0.252
T1	186 (54.4%)	94 (58.4%)	92 (50.8%)		144 (56.5%)	42 (48.3%)		41 (60.3%)	145 (52.9%)	
T2	128 (37.4%)	52 (32.3%)	76 (42.0%)		89 (34.9%)	39 (44.8%)		26 (38.2%)	102 (37.2%)	
T3	18 (5.3%)	10 (6.2%)	8 (4.4%)		15 (5.9%)	3 (3.5%)		1 (1.5%)	17 (6.2%)	
T4	7 (2.1%)	5 (3.1%)	2 (1.1%)		5 (2.0%)	2 (2.3%)		0 (0.0%)	7 (2.6%)	
Tumor stage										
0	3 (0.9%)	0 (0.0%)	3 (1.7%)	chi^2^ = 13.19, *p* = 0.040	2 (0.8%)	1 (1.2%)	chi^2^ = 3.96, *p* = 0.682	0 (0.0%)	3 (1.1%)	chi^2^ = 4.67, *p* = 0.586
IA	120 (35.1%)	64 (39.8%)	56 (30.9%)		95 (37.3%)	25 (28.7%)		27 (39.7%)	93 (34.0%)	
IB	62 (18.1%)	22 (13.7%)	40 (22.1%)		42 (16.5%)	20 (23.0%)		15 (22.1%)	47 (17.2%)	
IIA	43 (12.6%)	18 (11.2%)	25 (13.8%)		34 (13.3%)	9 (10.3%)		8 (11.8%)	35 (12.8%)	
IIB	42 (12.3%)	16 (9.9%)	26 (14.4%)		31 (12.2%)	11 (12.6%)		6 (8.8%)	36 (13.1%)	
IIIA	65 (19.0%)	36 (22.4%)	29 (16.0%)		46 (18.0%)	19 (21.8%)		12 (17.7%)	53 (19.3%)	
IIIB	7 (2.1%)	5 (3.1%)	2 (1.1%)		5 (1.0%)	2 (2.3%)		0 (0.0%)	7 (2.6%)	
Surgical treatment										
Bi-, Lobectomy (incl. Sleeve technique)	315 (92.1%)	150 (93.2%)	165 (91.2%)	chi^2^ = 0.47, *p* = 0.492	234 (91.8%)	81 (93.1%)	chi^2^ = 0.16, *p* = 0.689	66 (97.1%)	249 (90.9%)	chi^2^ = 2.86, *p* = 0.091
Pneumonectomy	27 (7.9%)	11 (6.8%)	16 (8.8%)		21 (8.2%)	6 (6.9%)		2 (2.9%)	25 (9.1%)	
Additional treatment										
Neo-adjuvant Chemotherapy	44 (12.9%)	21 (13.0%)	23 (12.7%)	chi^2^ = 0.01, *p* = 0.926	35 (13.7%)	9 (10.3%)	chi^2^ = 0.66, *p* = 0.416	4 (5.9%)	40 (16.6%)	chi^2^ = 3.69, *p* = 0.055
Adjuvant Chemotherapy	93 (27.2%)	33 (20.5%)	60 (33.2%)	chi^2^ = 6.89, *p* = 0.009	73 (28.6%)	20 (23.0%)	chi^2^ = 1.04, *p* = 0.307	12 (17.7%)	81 (29.6%)	chi^2^ = 3.91, *p* = 0.048
Adjuvant Chemo-Radiotherapy	11 (3.2%)	4 (2.5%)	7 (3.9%)	chi^2^ = 0.52, *p* = 0.469	8 (3.1%)	3 (3.5%)	chi^2^ = 0.02, *p* = 0.887	2 (2.9%)	9 (3.3%)	chi^2^ = 0.02, *p* = 0.886
Adjuvant Radiotherapy	34 (9.9%)	15 (9.3%)	19 (10.5%)	chi^2^ = 0.13, *p* = 0.716	24 (9.4%)	10 (11.5%)	chi2 = 0.31, *p* = 0.575	6 (8.8%)	28 (10.2%)	chi^2^ = 0.12, *p* = 0.731

**Table 3:** Causes of death of 342 patients undergoing curative resection for NSCLC. Abbreviations: NSCLC: non-small cell lung cancer, COPD: Chronic obstructive pulmonary disease.

Cause of Death	Number	Percentage (%)
NSCLC	157	45.9
Neoplasia other than NSCLC	22	6.4
Other than neoplasia	66	19.3
Chronic cardiac failure	11	3.2
Pneumonia	9	2.6
Myocardial infarction	8	2.3
Renal failure	7	2.0
Stroke	7	2.0
Dementia	5	1.5
Right heart failure	5	1.5
COPD	3	0.9
Decrepitude	2	0.6
Pulmonary embolism	2	0.6
Ileus	2	0.6
Parkinson´s disease	1	0.3
Antibody deficiency syndrome	1	0.3
Multiorgan failure	1	0.3
Peritonitis	1	0.3
Influenza	1	0.3
Total	245	71.6

**Table 4:** Univariable and multivariable analyses between OS, CSS and RFS and clinic-pathological characteristics of 342 patients undergoing curative resection for NSCLC. Abbreviations: OS: overall survival, CSS: cancer specific survival, RFS: recurrence free survival, COPD: Chronic obstructive pulmonary disease, NSCLC: non-small cell lung cancer, BMI: Body Mass Index, T: T stage (tumor invasion), UICC: International Union against Cancer, kg: kilogram.

	Univariable			Multivariable		
	Hazard Ratio	z	*p*	Hazard Ratio	z	*p*
OS						
Age	1.020 ± 0.006	2.99	0.003	1.026 ± 0.007	3.67	0.000
Weight	0.994 ± 0.004	−1.32	0.187			
Bmi	0.976 ± 0.016	−1.41	0.157	0.954 ± 0.015	−2.80	0.005
Female	0.088 ± 0.109	−1.63	0.103			
Smoking	1.075 ± 0.170	0.46	0.647			
Copd	1.013 ± 0.129	0.11	0.914			
Mucostasis	1.672 ± 0.234	3.67	0.000	1.703 ± 0.243	3.73	0.000
Adenocarcinoma	ref.					
Squamous cell carcinoma	1.088 ± 0.164	0.56	0.575			
Other	1.398 ± 0.221	2.12	0.034			
Grading	1.192 ± 0.112	1.86	0.063			
pT	1.464 ± 0.133	4.18	0.000			
Stage (UICC)	1.756 ± 0.139	7.08	0.000	1.679 ± 0.141	6.17	0.000
Pneumonectomy	1.682 ± 0.393	2.23	0.026			
Neo-adjuvant chemotherapy	2.243 ± 0.395	4.59	0.000	1.815 ± 0.348	3.10	0.002
Adjuvant chemotherapy	0.959 ± 0.139	−0.28	0.778			
Adjuvant chemo-radiotherapy	1.135 ± 0.408	0.35	0.723			
Adjuvant radiotherapy	1.406 ± 0.287	1.67	0.095			
CSS						
Age	1.008 ± 0.007	1.04	0.299	1.019 ± 0.008	2.28	0.023
Weight	0.989 ± 0.005	−1.91	0.056			
Bmi	0.966 ± 0.018	−1.75	0.081	0.954 ± 0.019	−2.30	0.021
Female	1.004 ± 0.155	0.03	0.979			
Smoking	0.923 ± 0.164	−0.45	0.655			
Copd	0.094 ± 0.133	−0.74	0.458			
Mucostasis	1.393 ± 0.237	1.95	0.052	1.588 ± 0.278	2.64	0.008
Adenocarcinoma	ref.					
Squamous cell carcinoma	0.772 ± 0.144	−1.37	0.169	0.619 ± 0.119	−2.49	0.013
Other	1.446 ± 0.254	2.10	0.036	1.213 ± 0.220	1.07	0.286
Grading	1.156 ± 0.127	1.32	0.187			
pT	1.438 ± 0.153	3.41	0.001			
Stage (UICC)	1.937 ± 0.177	7.22	0.000	1.873 ± 0.181	6.46	0.000
Pneumonectomy	1.424 ± 0.426	1.18	0.237			
Neo-adjuvant chemotherapy	2.664 ± 0.517	5.05	0.000	1.823 ± 0.402	2.72	0.006
Adjuvant chemotherapy	1.175 ± 0.191	0.99	0.323			
Adjuvant chemo-radiotherapy	1.560 ± 0.565	1.23	0.219			
Adjuvant radiotherapy	1.841 ± 0.397	2.83	0.005			
RFS						
Age	1.001 ± 0.007	0.16	0.869			
Weight	0.987 ± 0.005	−2.43	0.015			
Bmi	0.960 ± 0.017	−2.14	0.032	0.956 ± 0.017	−2.36	0.018
Female	1.110 ± 0.162	0.72	0.472			
Smoking	0.909 ± 0.154	−0.56	0.576			
Copd	0.944 ± 0.134	−0.40	0.688			
Mucostasis	1.371 ± 0.220	1.96	0.049	1.544 ± 0.255	2.63	0.009
Adenocarcinoma	ref.					
Squamous cell carcinoma	0.789 ± 0.137	−1.36	0.174	0.627 ± 0.104	−2.80	0.005
Other	1.333 ± 0.226	1.70	0.089			
Grading	1.123 ± 0.116	1.13	0.258			
pT	1.384 ± 0.137	3.27	0.001			
Stage (UICC)	1.794 ± 0.156	6.71	0.000	1.737 ± 0.160	5.99	0.000
Pneumonectomy	1.804 ± 0.471	2.26	0.024			
Neo-adjuvant chemotherapy	2.354 ± 0.451	4.46	0.000	1.637 ± 0.338	2.39	0.017
Adjuvant chemotherapy	1.290 ± 0.197	1.66	0.096			
Adjuvant chemo-radiotherapy	1.610 ± 0.550	1.40	0.163			
Adjuvant radiotherapy	1.638 ± 0.345	2.34	0.019			

## Data Availability

Data will be provided on reasonable request.
